# Phagocytosis by the Retinal Pigment Epithelium: Recognition, Resolution, Recycling

**DOI:** 10.3389/fimmu.2020.604205

**Published:** 2020-11-13

**Authors:** Whijin Kwon, Spencer A. Freeman

**Affiliations:** ^1^ Program in Cell Biology, Peter Gilgan Centre for Research and Learning, Hospital for Sick Children, Toronto, ON, Canada; ^2^ Department of Biochemistry, University of Toronto, Toronto, ON, Canada

**Keywords:** cholesterol, integrins, actin cytoskeleton, MerTK, glucose transport, V-ATPase, resolvins, phosphatidylserine (PtdSer)

## Abstract

Tissue-resident phagocytes are responsible for the routine binding, engulfment, and resolution of their meals. Such populations of cells express appropriate surface receptors that are tailored to recognize the phagocytic targets of their niche and initiate the actin polymerization that drives internalization. Tissue-resident phagocytes also harbor enzymes and transporters along the endocytic pathway that orchestrate the resolution of ingested macromolecules from the phagolysosome. Solutes fluxed from the endocytic pathway and into the cytosol can then be reutilized by the phagocyte or exported for their use by neighboring cells. Such a fundamental metabolic coupling between resident phagocytes and the tissue in which they reside is well-emphasized in the case of retinal pigment epithelial (RPE) cells; specialized phagocytes that are responsible for the turnover of photoreceptor outer segments (POS). Photoreceptors are prone to photo-oxidative damage and their long-term health depends enormously on the disposal of aged portions of the outer segment. The phagocytosis of the POS by the RPE is the sole means of this turnover and clearance. RPE are themselves mitotically quiescent and therefore must resolve the ingested material to prevent their toxic accumulation in the lysosome that otherwise leads to retinal disorders. Here we describe the sequence of events underlying the healthy turnover of photoreceptors by the RPE with an emphasis on the signaling that ensures the phagocytosis of the distal POS and on the transport of solutes from the phagosome that supersedes its resolution. While other systems may utilize different receptors and transporters, the biophysical and metabolic manifestations of such events are expected to apply to all tissue-resident phagocytes that perform regular phagocytic programs.

## Introduction

Phagocytosis, the ingestion of large (>0.5 um) particles, is an evolutionarily conserved, actin-driven process with roles in nutrient acquisition, immunity, and tissue homeostasis (1–3). The ongoing, routine phagocytic programs that maintain tissue homeostasis in the absence of infection or injury are largely performed by tissue-resident phagocytes. These cells are non-migratory and are therefore strategically positioned for phagocytic encounters. In some cases, resident phagocytes are uniformly distributed to optimally survey the whole tissue by probing the space between them (4, 5). In other cases, they are localized to particular regions that favor their collection and engulfment of phagocytic loads [e.g. splenic red pulp macrophages that turnover red blood cells (6) or bone marrow-resident macrophages that remove nuclei extruded during erythropoiesis (7, 8)]. In all cases, the efficient removal of dead cells, parts of cells, and debris by these phagocytes is essential to prevent secondary necrosis, inflammation, and autoimmunity (9, 10).

**Figure 1:**
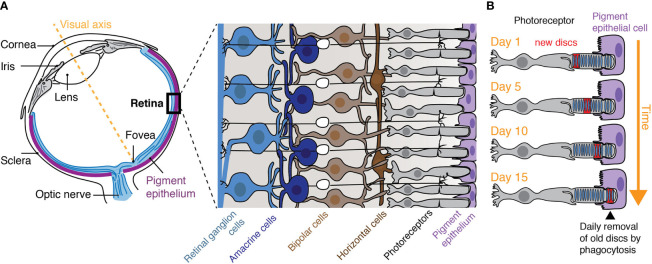
The retinal pigment epithelium. **(A)** The retina lines the back of the eye and is comprised of five cell types: rod and cone photoreceptors, bipolar, horizontal, amacrine, and retinal ganglion cells. **(B)** The retinal pigment epithelium that underlies the retina is responsible for the turnover of photoreceptor outer segments (POS) by their phagocytosis and is critical for photoreceptor function. Photoreceptors are choc-a-bloc with membrane discs that harbor opsins which are susceptible to phototoxic damage. The most distal segments of the POS that contain the oldest discs are therefore removed from the live photoreceptors while new discs are made at the beginning of the outer segment. Note: as new discs are formed at the base of the POS, old discs are removed by phagocytosis, a process that takes approximately 2 weeks.

Within the category of tissue resident phagocytes are “specialized phagocytes”; epithelial derived stromal cells including the retinal pigment epithelium (RPE) of the eye and Sertoli cells of the testes (10, 11). As epithelia, these cells contribute to the formation of blood-tissue barriers while facilitating the directional transport of oxygen, glucose, cholesterol, etc. to the tissue from circulation. As specialized phagocytes, these cells also actively turnover theirs neighbors. Perhaps the best studied example of specialized phagocytes is indeed the RPE that intimately associates with photoreceptors and mediates their turnover (12). A single RPE cell is in contact with ~30 photoreceptor cells (rods and cones) and is responsible for the phagocytosis and removal of the distal portions of the photoreceptor outer segments (POS) that are phagocytosed in a diurnal fashion (13, 14) (**Figure 1**). The removed mass is not trivial; 7–10% is eliminated daily meaning the entire POS population is turned over every 2 weeks (15). Remarkably, a burst of phagocytosis is timed with the entry of light to the retina and, within hours, the phagosomes formed in each healthy RPE cell are resolved (14, 16).

Expectedly, the removal of the aged regions of the POS and of metabolic wastes by the RPE is essential for the homeostasis of the retina (17, 18). Just as critically, the ingested material by the RPE needs to be resolved otherwise it accumulates and forms lipofuscin (comprised of oxidized proteins and lipids), eventually leading to retinal disorders (19). Lipofuscin also accumulates in the eye with healthy aging (20, 21), so an understanding of when and how this causes disease is fundamental. RPE cells are themselves post-mitotic and therefore resolution of the vacuoles formed during phagocytosis is integral to RPE longevity. The resolution process can be envisaged as a sequence of steps that includes the maturation of the nascent phagosome, the digestion of luminal macromolecules, the efflux of solutes from the phagolysosomes, and the resorption of the phagosomal membrane. Interestingly, the building blocks from the ingested photoreceptor outer segments (POS) are thought to be shuttled back to the photoreceptor cells (22), supporting their continuous regeneration and completing a “heterocellular metabolic circuit.” Importantly, in the RPE-photoreceptor relationship, it is appreciated that dysfunction in one cell type leads to degeneration in the other. Such metabolic coupling and the sequence of events that complete these circuits, while recognized in the case of the RPE and photoreceptors, are poorly understood for other tissue-resident phagocytes (23).

Here we describe the phagocytosis, breakdown, and resolution of ingested POS by RPE cells. We first illustrate phagocytosis by the RPE including the binding, ensheathment, and ingestion of the POS driven by the RPE actin cytoskeleton. We subsequently describe the maturation steps of the phagosome that confer on the vacuole its degradative capacity. The eventual efflux of solutes from the phagolysosome is discussed in some detail and we describe how these fluxes are expected to lead to the remodeling of the phagosomal membrane including its vesiculation and tubulation. Finally, we propose that the delivery of building blocks back to the tissue microenvironment supports the health of neighboring cells and we speculate on how such events may occur.

### Photoreceptors and the Retinal Pigment Epithelium: Close Encounters

Light that enters the vertebrate eye is focused onto the neural retina, a thin tissue (∼200 µm) comprised of five main classes of cells including photoreceptors, bipolar cells, amacrine cells, horizontal cells, and ganglion cells (**Figure 1**). These cells work concertedly to process and transmit visual information to the midbrain, thalamus, and visual cortex *via* the optic nerve (24). Despite their posterior positioning (e.g. at the back layer of the retina and the region farthest from incoming light), it is with the photoreceptor cells where light is captured and the visual pathway is initiated. The light is entirely absorbed in outer segments of the photoreceptor. The POS consists of membranous discs packed with integral membrane proteins called opsins. Opsins are made to be light-sensitive by their covalent association with the chromophore retinal (e.g. rhodopsin in the case of rods) (25) and are perhaps the best studied of the G-protein coupled receptors. Upon light exposure, rhodopsin undergoes immediate bleaching which triggers the visual transduction pathway. Over time, and with repetitive bleaching and regeneration of rhodopsin, phototoxic damage can occur in the neighboring proteins and lipids of the discs (26). To circumvent the accumulation of damaged components, photoreceptors undergo continuous turnover facilitated by the synthesis and assembly of new discs at the base of the outer segment and the simultaneous shedding of the oldest discs from the growing tips of the POS (**Figure 1**). This mechanism results in photoreceptors that are long-lived and discs of the POS that are short-lived (~2 weeks).

**Figure 2:**
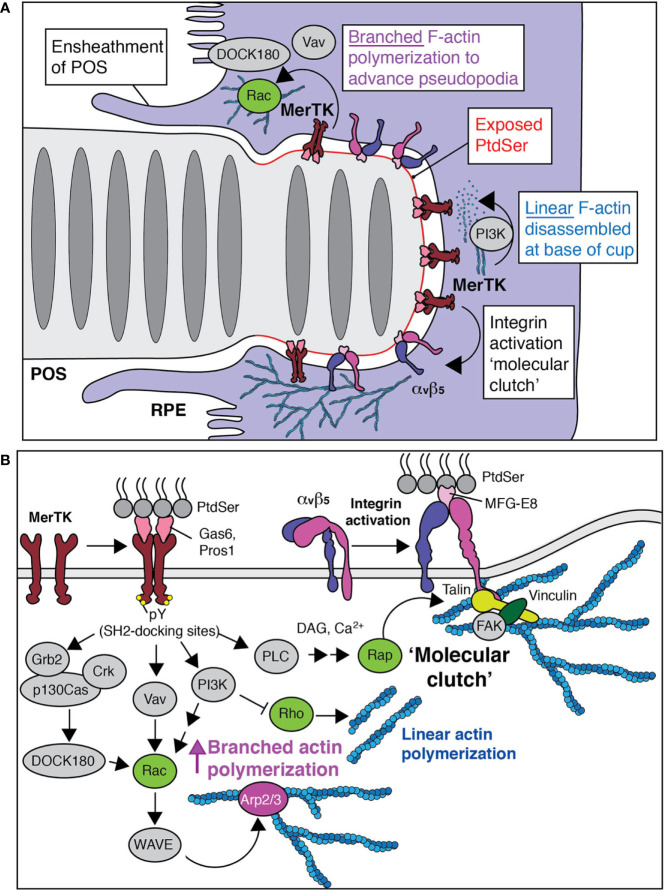
Phagocytosis by the retinal pigment epithelium. **(A)** Like all types of phagocytosis, the engulfment of POS by the RPE is driven by actin polymerization. PtdSer is first exposed and tethered by bridging molecules that bind the distal portion of the POS and MerTK receptors. To relieve membrane tension, linear networks are removed by the activation of PI3K. Branched networks are stimulated by nucleation promoting factors that bring actin monomers to the Arp2/3 complex. Both activities are mediated by MerTK (see also panel **B)**. **(B)** MerTK initiates a series of signaling pathways that stimulate branched actin polymerization, networks that attach to the membrane *via* integrins. The major RPE integrin is αvβ5, which also binds to PtdSer but *via* MFG-E8. As actin advances the pseudopodia tips of the RPE and facilitates severing of the POS, clearance of actin from the base of the cup allows for the delivery of new membrane, a PI3K-dependent process.

At the tip of the POS facing the RPE, is the marked accumulation of phosphatidylserine (PtdSer) exposed on the outer leaflet of the cell which becomes much more pronounced with light onset (16) (**Figure 2**). In virtually all other healthy cell types, the asymmetric distribution of PtdSer to the inner leaflet is tightly maintained by the ongoing activity of phospholipid translocases or “flippases” including ATP11A and ATP11C (27–30). Only under apoptotic conditions are these flippases cleaved and inactivated by caspases. The same caspases also cleave and activate scramblases, which can alternatively be activated by Ca^2+^, that begin to randomly flip membrane phospholipids like PtdSer between the two leaflets (31–34). Given the tight control over the asymmetric distribution of PtdSer in most cells, the polarized and relatively sustained exposure of the phospholipid in the non-apoptotic photoreceptor is a unique phenomenon. It must require 1) the very local disruption of flippases/activation of scramblases and 2) a barrier to the free diffusion of exofacial PtdSer from the distal POS tip.

The latter may be achieved by secreted molecules that bridge and tether the PtdSer to RPE receptors. Plenty of bridging molecules may participate in trapping the exposed PtdSer including Gas6, Protein S, Tubby, and Tubby-like protein 1 which connect PtdSer to the RPE-expressed MerTK receptor (35–38) and MFG-E8 which does the same for αvβ5 integrins (39). Given that exposed PtdSer remains polarized to the distal tips of POS in *Mfge8*-/- and *Itgb5*-/- (β5 integrin) mice (16), there may be functional redundancy to this effect or the MerTK ligands may be the essential factors. Here, at least Gas6 and Protein S are provided to the interphotoreceptor matrix by their synthesis and release from the RPEs themselves (35, 37, 40). At least some of these bridging molecules were recently shown to be transcribed by the RPE in a circadian fashion (41). Fittingly, experiments challenging RPEs with POS *in vitro* are often performed by augmenting the culture with exogenous Gas6 and/or Protein S (42) suggesting their requirement and some mechanism(s) of regulation to their expression by light.

How the local inactivation or exclusion of flippases and activation of scramblases at the distal tip of the POS occurs and is regulated in a diurnal fashion, on the other hand, remains entirely unclear. Notably, scramblases of the anoctamin/TMEM16 family show a polarized localization in other cell types (43) and their activation causes membrane expansion and shedding (44). A role for local Ca^2+^ flux and caspase activation has also been suggested (27).

### Photoreceptors and the Retinal Pigment Epithelium: Phagocytosis

The association between the POS of the photoreceptor and the RPEs is constant: The apical microvilli of the RPE probe and become elaborated to “ensheath” the outer segments as they accumulate PtdSer, long before their phagocytosis (**Figure 2**) (45–47). Such membrane projections from the RPE can reach nearly half-way up the POS (up to ~15 µm), yet little is known about the cytoskeleton and the associated membrane remodeling that supports these structures. It is unlikely to be mediated by the features shared between the RPE microvilli and those found in other epithelial cells like ezrin/radixin/moesin (ERM) proteins (45, 48) and ERM-binding proteins including NHERF1 (49), which tend to oppose broad ruffling events (50, 51). Other features of the RPE apical membrane including the Na^+^/K^+^-ATPase pump, while also unique, are unlikely to directly drive ensheathment.

Instead, it is known that the ensheathment of POS requires the engagement of apically targeted MerTK, a member of the Tyro/Axl/Mer family of receptor tyrosine kinases (52, 53). Once MerTK is engaged by Gas6 or Protein S, ligand binding induces its multimerization and trans-autophosphorylation by the kinase domain. This leads to the recruitment of a number of adaptors and effectors that facilitate pseudopod extension (**Figure 2**). For example, the phosphorylated tyrosines in the cytosolic aspect of the receptor serve as docking sites for Vav, a potent GEF for Rac GTPases (54). The ensheathment structures indeed resemble those of spontaneous ruffles that are observed in macrophages (55) or that can be stimulated by pathogens (56) in a manner dependent on the activation of Rac GTPases. MerTK also stimulates Rac activation by recruiting the p130Cas/CrkII/Dock180 GEF complex (57). Rac-GTP can then bind and activate the WASP-family verprolin-homologous protein (WAVE) regulatory complex (WRC), (58, 59) which functions as a nucleation promoting factor for Arp2/3-branching of the F-actin cytoskeleton. Though less appreciated in recent literature, F-actin branching has long been known to also be mediated by flexible dimers of filamins including filA (60). No matter how they are formed, heavily branched networks of F-actin are indeed associated with broad membrane ruffling events akin to those of ensheathing RPEs (61). That Rac activation is associated with ensheathment is supported by data demonstrating Rac is indeed essential for the phagocytosis of POS (62).

MerTK also recruits and activates PI3K which in turn phosphorylates PtdIns(4,5)P_2_ to generate PtdIns(3,4,5)P_3_ a phosphoinositide that can subsequently be converted to PtdIns(3,4)P_2_ (63). PtdIns(3,4,5)P_3_ and potentially PtdIns(3,4)P_2_ support Rac activation by recruiting Rac GEFs and also dismantle linear actin networks by recruiting Rho specific GAPs (64) (**Figure 2**). The latter is critical for removing the submembrane highly bundled actin cables of the cortex that allows for the focal delivery of membrane from the endocytic pathway. Collectively, these actions relieve membrane tension that opposes membrane ruffling (65–68) and helps to explain why PI3 kinases are critical for the phagocytosis of large particles but not small ones (69, 70). While the phagosomes formed by the RPE are only 1-2 µm in diameter, a single RPE needs to engulf ~30 targets at once—a number that can in fact be much higher in the center of the retina—while maintaining junctions with its neighbors. Given the amount of new membrane delivered and/or the unfurling of the microvillar membrane that would require substantive F-actin disassembly for ensheathment, it makes sense that PI3K activation is indeed a prerequisite for synchronized POS phagocytosis and that its inhibition leads to increased F-actin at aborted phagocytic cups (71). That Rho inactivation is central to the process is supported by experiments demonstrating decreased phagocytosis when Rho kinase is made to be constitutively active (72).

While branched networks of F-actin propel and advance broad regions of the plasma membrane, they must be stabilized and connected to adhered transmembrane proteins to prevent the retrograde flow and collapse of the entire effort (**Figure 2**). Such points of stability are described as “molecular clutches” that limit the slipping of the branched F-actin networks (73) and yield efficiency to phagocytosis in many contexts (74, 75). A molecular clutch in the RPE membrane could be facilitated by the binding of the RPE cadherins to the neural cell adhesion molecule (N-CAM) expressed in the POS (76). Interestingly, aged whole animal KO *Ncam* ^-/-^ mice have thinned photoreceptor cell layers and premature vision-loss (77). A specific role for N-CAM in the RPE has not been identified but is well-expressed in these cells. Another obvious molecular clutch for the ruffling RPEs is the major RPE integrin, αvβ5 which is targeted apically. A role for the integrin here would explain why ensheathment is not stimulated by peppering the RPEs with MFG-E8 alone (45) but that the efficient, diurnal removal of the portions of the POS indeed requires the integrin and its ligand (16, 78).

### Crosstalk Between MerTK and αvβ5

The MerTK and αvβ5 pathways are not mutually exclusive (**Figure 2**) (57). Like other integrins, αvβ5 is thought to exist primarily in a “bent” or closed conformation with low affinity for ligand (79). Its inside-out activation is triggered by receptor tyrosine kinases like MerTK. Once MerTK is engaged, a number of SH2-containing proteins are then recruited to the phosphorylated tyrosine residues including the adaptors Vav, Crk, and Grb2 as well as the phospholipase C gamma (PLC*γ*) (80–82) (**Figure 2**). Importantly, these multi-molecular assemblies favor the recruitment and activation of Rap GTPases which in turn recruit the proteins necessary to disrupt the inactive conformation of the integrin including the Rap1-GTP-interacting adapter molecule (RIAM) and Talin (83) leading to its inside-out activation. Talin, once bound to the β5 subunit of the integrin heterodimer, provides binding sites for F-actin and F-actin-binding proteins like vinculin.

Such an effect is critical in the case of MerTK which itself does not bind F-actin directly or even, to our knowledge, indirectly. Since integrins provide the mechanical linkage between actin polymerization to the ruffling/ensheathing membrane to yield traction, it makes sense that their activation by primary phagocytic receptors is observed in many types of phagocytosis (84). In this regard, it is noteworthy that MerTK-mediated phagocytosis involves focal adhesion kinase (FAK) (85), regulation of FAK activity (62), and the cleavage of PtdIns(4,5)P_2_ to generate Ins3P/diacylglycerol (DAG), which are normally associated with integrin and Rap activation respectively (86). Still, and as previously described, other ancillary transmembrane clutches are possible. Just as integrins can augment MerTK initiated phagocytosis, other receptors also participate including Tyro3 of the Tyro/Axl/Mer family of receptor tyrosine kinases (87) and the scavenger receptor CD36 (88). The relative contribution of Tyro3 and CD36 is still unclear and MerTK remains the canonical phagocytic receptor of the RPE; only the ablation of MerTK in the RPE leads to severe photoreceptor degeneration.

### Sealing of the Nascent RPE Phagosome

The final steps of phagocytosis—sealing of the nascent phagosome by fusion of the pseudopodia tips—is the least understood step in all instances of engulfment and is especially poorly understood in the case of the RPE. This is remarkable since the RPE must apply a sufficient amount of local force on the POS to deform and generate scission through two lipid bilayers of an intact, live cell. The nascent phagosomes formed by RPE cells are indeed regular in their size (1–2 µm in diameter) as observed by electron (18) and light microscopy (89), as would be expected given the regular turnover of the POS, so the steps leading to scission must also be regulated. The site of eventual scission may be demarcated on the POS by the polarized distribution of PtdSer, which expands just before the scission event (16). However, the final, inward constricting force to complete phagocytosis remains enigmatic. In other systems, this has been attributed to the dynamin machinery employed in generic forms of endocytosis (90) and this could also be the case for the RPE. Alternatively, or in addition to dynamin, myosins may provide a “purse-string” constricting force as has been observed during the phagocytosis of targets by macrophages (91). Impressively, such a constricting force is sufficient to deform the skeleton of red cells (91) and to remove parts of live cells during ‘trogocytosis” (92). Indeed, MerTK activation in the RPE leads to the marked recruitment of Myosin II-A and Myosin II-B to the phagocytic cup, and the pharmacological or genetic ablation of Myosin II partially prevents POS engulfment *in vitro* (93). However, like the majority of the systems used to investigate RPE phagocytosis, here the targets were delivered in a pre-severed state. Clearly, further studies are necessary to visualize these events and to determine the mechanism(s) involved.

### Phagosome Maturation

**Figure 3:**
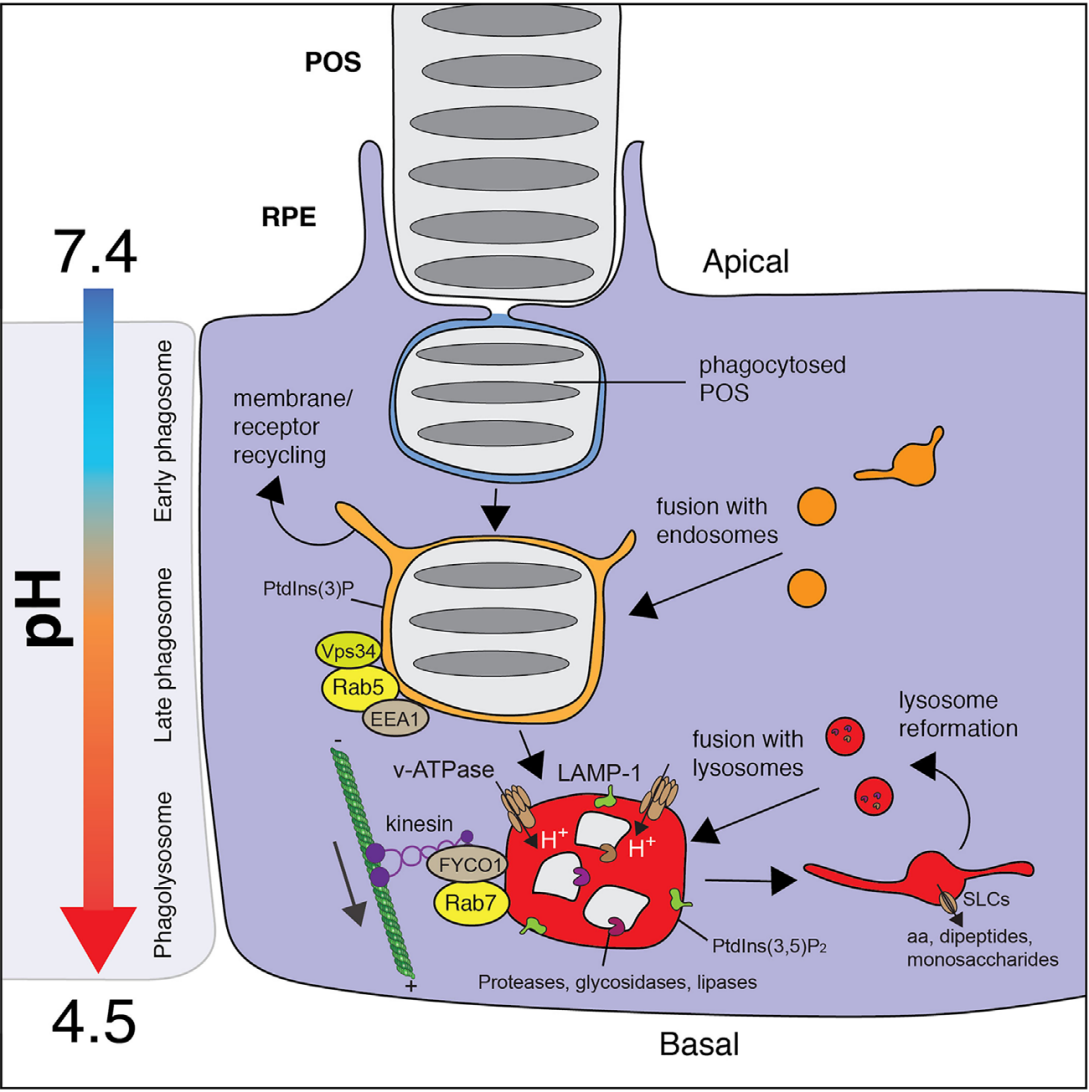
Maturation of the RPE phagosome. Internalized POS particles are sealed in a nascent phagosome that undergoes step-wise maturation which involves the graded fusion of endosomes and then lysosomes. The maturation of the phagosome is delineated by its association with Rab GTPase and its phosphoinositide constituents, which act to coordinate fusion. Maturation also involves microtubule-based movement toward the cell center, a switch in Rabs [Rab5 → Rab7] and phosphoinositide [PI(3)P → PI(3,5)P_2_] composition, a drop in pH [6.5 → 4.0], and the activation of phagosomal proteases. In the RPE, the movement of the phagosome is dependent on kinesins which traverse the cytosol towards the plus ends of the microtubules on the basal side of the cell. Once solutes are transported from the phagolysosome, the compartment undergoes fragmentation to reform and replenish the pool of lysosomes.

Following its scission from the plasma membrane, the nascent RPE phagosome undergoes graded fusion with endosomes and then lysosomes in a complex process termed “maturation” (**Figure 3**). Maturation can be envisaged as a series of steps marked by changes in the signaling phosphoinositides found within the limiting membrane of the phagosome and changes in its association with Rab GTPases in particular (12, 94). Importantly, maturation results in the acquisition of the V-ATPase and the delivery of enzymes from lysosomes that grant the vacuole its degradative properties. Maturation also coincides with—and is highly dependent on—the motor-driven movement of the phagosomes toward the basolateral side of the epithelial cell where they collect in a juxtanuclear location (95, 96). While the movement of the POS-containing phagosome through the cytosol differs markedly from the phagosomes formed in myeloid cells, the maturation process otherwise shares many of its salient molecular features. In this section, we therefore describe maturation in general terms and highlight what is known in the RPE system.

The Rab family of GTPases that orchestrate membrane fusion events and organellar trafficking are indispensable for maturation (94). Following its sealing, Rabex-5, a guanine nucleotide exchange factor (GEF), is targeted to the early phagosome to recruit and activate Rab5, Rab21, and Rab22 (97). While these Rab5 subfamily members all play important roles in phagosome maturation (98), Rab5 remains the best characterized member (99). Once active, Rab5 binds to its effectors including Rabaptin-5 which in turn re-stimulates Rabex-5, triggering a positive-feedback loop (100). Additionally, Rab5 exerts its function through the recruitment and activation of the phosphatidylinositol 3-kinase (PI3K) Vps34, which results in the rapid production of phosphatidylinositol 3-phosphate (PI3P). PI3P is necessary for the recruitment of early endosomal antigen 1 (EEA1), a protein that is essential for phagosome maturation by promoting its fusion with endosomes (99).

Rab5 and EEA1 are indeed reported to localize to the early RPE phagosome along with PI3P (101). Moreover, the conditional knockout of Vps34 leads to disordered phagosome trafficking, impaired lysosome fusion, and retinal degeneration in mice, supporting a similar role for PI3P in the RPE system (102). As the RPE phagosome matures, marked by its incorporation of LAMP proteins, the localization of Rab5 and EEA1 are expectedly depressed (101). Though mechanisms remain to be elucidated, the LAMP family of lysosome-resident glycoproteins is indeed required for the fusion of lysosomes with the phagosome (103). It is therefore noteworthy, that the loss of just one of these family members, LAMP2, in the murine RPE causes delayed POS degradation and the build-up of POS debris (104). In fact, the loss-of-function mutations in *LAMP2* that cause Danon disease in humans is also associated with retinal and macular degeneration (105).

In all cases, progression to the late phagosome is marked not just by the acquisition of LAMP proteins and its luminal acidification (described later), but also by the transition from being Rab5- to Rab7-positive, known as a Rab5-Rab7 “switch” (106). The sharp transition between Rab5 and Rab7 is explained by Rab7 GEFs that inhibit Rab5 activation. Specifically, Mon1, a subunit of the Rab7 GEF Mon1-Ccz1 that recruits and activates Rab7, also ousts Rabex-5 and arrests the Rab5 feedback loop (107). The Rab5-Rab7 transition promotes the subunit substitution necessary for the transformation of CORVET, a protein complex associated with early endosomal fusion, to HOPS, associated with later stage endosomal fusion (108).

In addition to facilitating fusion with late endosomes and lysosomes, Rab7 activation also coordinates the inward movement of the phagosome. In myeloid cells, the Rab7 effectors Rab7-interacting lysosomal protein (RILP) (109) and OSBP-related protein 1 (ORP1L) (110) form complexes that cluster dynein motors on the phagosome to cooperatively facilitate retrograde movement (111). The apical to basal movement of phagosomes in the RPE, while indeed dependent on Rab7 and microtubules (112, 113), differs markedly. First, the phagosomes formed by the RPE are initially engaged by Myosin VIIa, an unconventional myosin that moves along F-actin. Myosin VIIa is of particular interest since mutations in the MYO7A gene cause Usher syndrome 1B which involves progressive retinal degeneration (114). Fittingly, Myosin VIIa is largely expressed in the apical region of the RPE where it may facilitate the initial transport of the phagosome through a dense apical F-actin network until it can latch on to microtubule motors (96, 115). Second, the RPE phagosomes are then bound by kinesin motors and their associated kinesin-1 light chain 1 (KLC1), rather than dynein. The RPE phagosomes on the apical side of the cell lose their association with Myosin-VIIa as they begin to associate with KLC1, an event that coincides with their movement toward the basolateral side (95, 116). Mice lacking KLC1 show delayed phagosome progression to the basal region of the cell, the accumulation of debris and drusen, and ultimately the loss of photoreceptors (96, 116). In contrast to myeloid cells, a strong association with dynein motor complexes is in fact associated with delayed phagosome maturation and impaired phagosome motility in RPE (95). The obvious difference in phagosomal transport through the cytosol between cell types is attributed to the polarity of the microtubule tracks that are inversely oriented in the RPE (12): The plus ends of the microtubules are found at the basolateral side of the RPE. Rab7-GTP on the RPE phagosome must therefore mediate connections between the organelle and kinesins. Interestingly, active Rab7 binds FYCO1 and kinesin light chain/KIF5 (117). A role for this complex in orchestrating phagosome motility in the RPE is unknown, though FYCO1 is known to at least control the positioning of lysosomes in RPE cells (118).

The precise regulation of Rabs by their posttranslational modification is also essential for phagosome maturation, especially in the RPE. For example, the ablation of the Rab escort protein (*Rep1*), a geranylgeranyltransferase that acts on Rab7 and others, causes a delay in phagosome maturation and POS clearance, the accumulation of debris in the RPE, and a pathology resembling age-related macular degeneration (119, 120). Clearly, the efficient mobilization and maturation of the RPE phagosome is critical for retinal health and homeostasis and has some unique features that warrant further study.

### Acidification of the Phagosome

As phagosomes form in the RPE every morning, their lumens acidify. The drop in pH is largely driven by the vacuolar ATPase (V-ATPase) that hydrolyzes ATP to pump protons into the lumen. Many of the digestive hydrolases, delivered to the phagosome by its fusion with endolysosomes, are then activated by the decrease in pH. That the acidification of the phagosome is indeed critical for the proper digestion of POS is exemplified by experiments demonstrating that inhibiting the V-ATPase in the RPE causes their accumulation of swollen phagolysosomes that do not degrade opsin proteins (121–123). This ultimately leads to the build-up of undigested debris between the Bruch’s membrane of the choroid and the RPE, a phenotype akin to age-related macular degeneration (AMD) in humans (12).

Despite the imperative role for acidification of the phagosome *via* the V-ATPase, it is a process that remains remarkably enigmatic in the RPE system. This is partly explained from an operational standpoint. Reliable measurements of phagosomal pH entail the selection of a suitable probe and the ability to target it to the phagosome. In the case of retinal flat mounts, phagocytic targets (i.e. POS) cannot be readily and specifically labeled. Most pH determinations of the RPE phagosome, and even of their lysosomes, have therefore been estimated by qualitative or semiquantitative means using predominantly membrane-permeant weak bases that accumulate in acidic organelles (89, 124). These include fluorescent dyes like LysoTracker (89), that accumulates in the phagosome without altering its fluorescence or LysoSensor that undergoes a pH-dependent spectral shift (124). While such sensors are acceptable indicators of acidity, they come with limitations (125). For example, when used at sufficiently high concentrations, membrane-permeant basic probes can themselves alter the pH of the (phago)lysosome. Nevertheless, LysoSensor has been used to determine that lysosomes in RPE cells in culture are pH 4.5 on average (124).

More recently, a genetically encoded chimera of an intralumenal, pH-sensitive GFP tethered to a cytosolic mCherry *via* a lysosomal transmembrane domain has been utilized to measure the pH of RPE lysosomes (126). Such a system also has caveats. First, the pKa of GFP is ~6.0 which is not optimal for determinations of the more acidic pH values expected within lysosomes and phagolysosomes. Second, the proteases within the lysosome are expected to degrade the internal GFP thereby conflating measurements of the proteolytic activity and the pH of the compartments.

Generally, the pH of the POS-containing phagosome or the rate at which it acidifies has not been formally assessed but has been inferred by the degradation of its contents (123) or by LysoTracker (89). Other, non-ratiometric dyes like rhodamine-based pHrodo^®^, that exhibit strong fluorescence in acidic environments have also been used to grossly estimate the pH of RPE phagosomes (127, 128).

The rates and extent of acidification should not be tacitly assumed to occur in all phagocytes similarly. One consideration is that in isolation, the V-ATPase driven proton influx required for acidification is self-limiting as it creates a membrane potential detrimental to the efficient acidification of the phagolysosome. Such an undesirable electrogenic consequence is circumvented by accompanied counterion fluxes that dissipate the building voltage. This can be achieved by cation efflux (e.g. the expulsion of Na^+^ or K^+^) or concurrent anion influx (e.g. of Cl^−^) (129) and these pathways may differ widely between cell types. Little is known about counterion flux mechanisms in any phagocyte, however, it is noteworthy that the whole animal loss of ClC7 or ClC3, chloride exchangers that can provide such counterion fluxes, results in retinal degeneration. While a direct role for ClCs in the RPE is not yet known, this phenotype could suggest that Cl- flux into the RPE phagosome may be critical for its acidification and breakdown of POS (130, 131).

Taken together, given that the rapid acidification supersedes enzymatic breakdown of the POS in the phagosome and is critical in maintaining retinal health, additional experiments should be done in this area. Clearly, a complete picture for the mechanisms that regulate the V-ATPase, pH, and maturation of the RPE phagosome has yet to emerge.

### Distilling and Breaking Down Macromolecules

The delivery of the V-ATPase and acidification of the phagosome is concomitant with the enzymatic breakdown of its contents (**Figure 3**). Though published results vary, as much as 70% of the POS dry mass is heavily glycosylated opsin, and the remaining 30% is polyunsaturated lipids, and therefore the proper digestion in the phagosome requires the delivery and activation of proteases, glycosidases, and lipases (132). The removal of water from the phagosome is also thought to be an important early step that facilitates membrane recycling, decreases the volume required for the pump to acidify, and increases the contact between enzymes and their substrates (133). This early step is initiated by monovalent ion efflux followed by outward movement of osmotically-obliged water (5) that presumably exits through aquaporins or other pores and channels that make mammalian membranes water-permeant.

The proteases involved in breaking down opsins which account for >95% of the protein content of the shed POS (134) into peptides and amino acids are primarily cathepsins. Cathepsin D, a ubiquitously expressed aspartyl protease, has garnered particular attention as it is both highly-expressed in the RPE and its genetic inactivation in mice leads to incompletely digested rhodopsins in the phagosome, the general accumulation of undigested phagocytic debris, and photoreceptor death (135, 136). There are numerous cathepsins that function as cysteine proteases in the RPE, on the other hand, so while these have also been suggested to play a role in proteolytic degradation of the OS target, there is likely functional redundancy between them (12). Making matters more complex, in addition to their autoactivation, aspartyl proteases like cathepsin D can be cleaved and activated by these cysteine proteases in the RPE lysosome (137).

The major phospholipids of the POS are phosphatidylcholine and phosphatidylethanolamine which account for ~80% of the total lipid, followed by phosphatidylserine (13%), and minor contributors like phosphatidylinositol and sphingomyelin (138). As the photoreceptor discs age, their cholesterol content tends to decrease, going from 30% of the molar lipid when first generated by their invagination from the plasma membrane to 10% when shed as part of the distal POS (139). Ingested photoreceptor lipids, including phospholipids and glycolipids, are broken down in the RPE phagolysosome by pH-dependent lipid hydrolases, namely phospholipase A_1_ and A_2_ (140). Cholesterol, on the other hand, is liberated from the POS and possibly de-esterified *via* the lysosomal acid lipase type A (141) and once soluble, transported into the limiting membrane of the phagosome. This requires a system of cholesterol-binding proteins that support movement of the hydrophobic molecule through tunnel-like structures. It can be achieved by the concerted action of Niemann–Pick type C (NPC) 1 and 2 (142) as well as by LIMP-2 (143). Niemann-Pick proteins are indeed necessary for normal retinal function (144). Upon its incorporation into the phagolysosomal membrane, cholesterol transport to extra-lysosomal destinations occurs through vesicular and non-vesicular routes (i.e. membrane contact sites). By these means, cholesterol can be delivered to lysosomes and/or trafficked to the plasma membrane, the Golgi apparatus and the endoplasmic reticulum. Remarkably, as discussed later, the efflux of POS-derived cholesterol back to the photoreceptor by the RPE appears to be a means for its reutilization.

As opsins are replete with glycans, a series of glycosidases are also deployed by the RPE to the phagosome including alpha-mannosidase, beta-glucouronidase and alpha fucosidase (145). Human diseases involving a deficiency of these enzymes commonly have retinal phenotypes, including degeneration, photoreceptor death, vision loss, and undigested phagocytic debris, suggesting that these enzymes play an important role in POS catabolism (146, 147).

Finally, the POS is in fact embedded within an interphotoreceptor matrix (IPM) that has a unique molecular composition that differs from other extracellular matrices. The major insoluble components of the IPM are in fact glycosaminoglycans (i.e. hyaluronic acid and proteoglycans with heparan, chondroitin, and keratan sulfate chains) (148). While the soluble fraction of the IPM contains only small amounts of these, the RPE is expected to take up portions of the IPM along with the POS during phagocytosis and may even facilitate the turnover of the IPM over time. It follows that deficiencies in the enzymes responsible for the breakdown of the glycosaminoglycans (mucopolysaccharidoses) have some sort of retinal phenotype (149).

### Solute Efflux and Resolution of the RPE Phagolysosome

Organic and inorganic solutes liberated or accumulated in the phagosome need to be effluxed from the vacuole in order for the compartment to “resolve” (**Figure 3**). Resolution is a normal part of the phagocytic process but remains the least understood aspect. In the case of the RPE, the return of the phagosomal membrane to replenish the pool of lysosomes is essential. Indeed, there are no observed circadian changes in the expression of prototypical lysosomal proteins in the RPE and so the pool of lysosomes used by the phagosomes must be returned to the cell (89).

**Figure 4:**
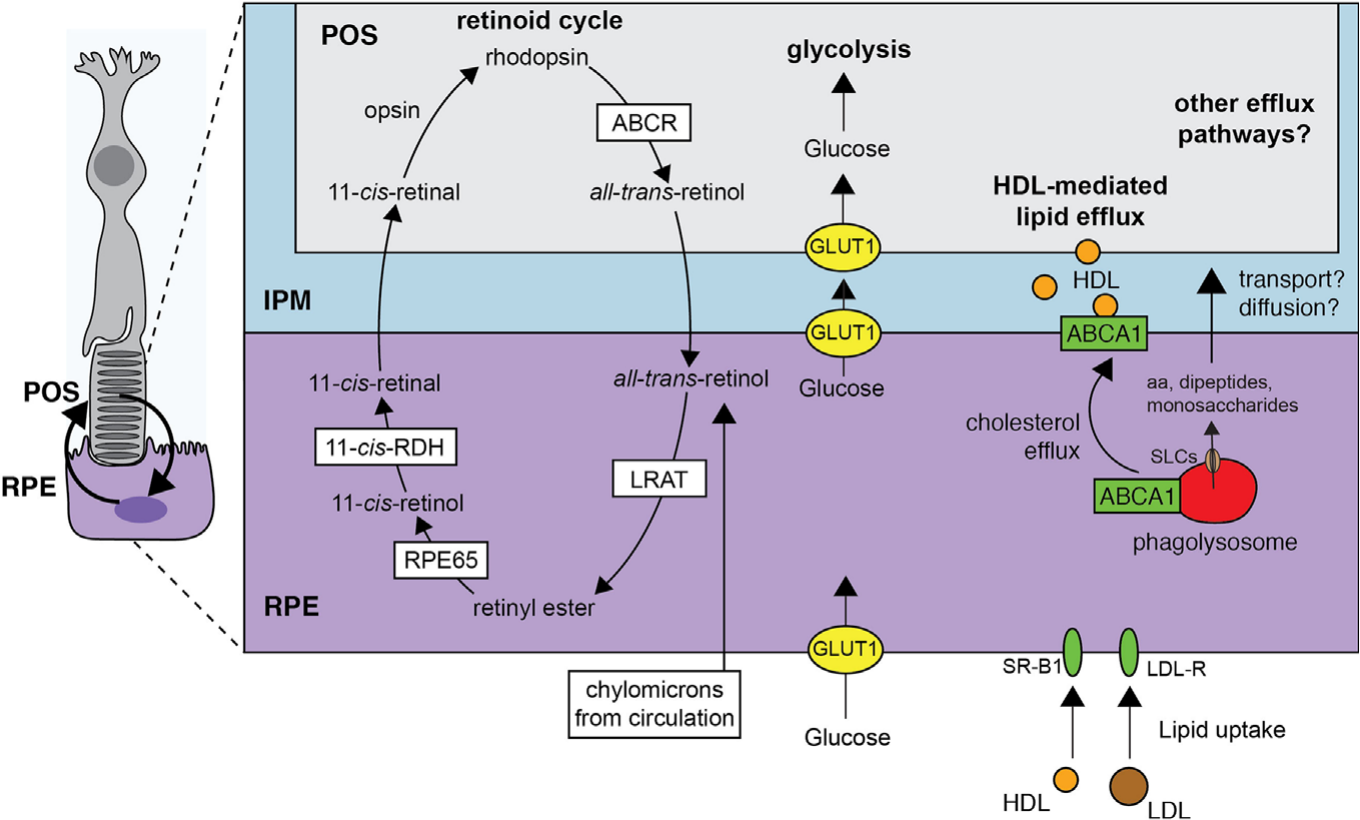
Digestion and solute efflux in the mature RPE phagolysosome. The retinoid cycle necessitates the active participation of the RPE. Upon photoisomerization of 11-cis-retinal to *all*-*trans*-retinal, the chromophore activates opsins and phototransduction. The removal of *all*-*trans*-retinal can then occurs by active means or passive diffusion but importantly, is delivered to the RPE for its esterification (catalyzed by lecithin:retinol acyltransferase/LRAT). Isomerization to 11-*cis*-retinol and oxidation to 11-*cis*-retinal also occurs in the RPE before diffusion back to the POS. The coupling between the POS and the RPE does not stop there. Glucose is transported to the interphotoreceptor matrix from circulation by the RPE and could conceivably also come from the phagolysosome. Similarly, high density lipoprotein mediates lipid efflux from the RPE to be used by the photoreceptors. Lipids, including cholesterol, are either transported from circulation or recycled from the POS-containing phagosome. Finally, other building blocks like amino acids may be supplied to the IPM by means not yet determined.

In the final stages of resolution, the efflux of solutes is in fact proposed as the mechanism that drives the reformation of lysosomes (**Figure 4**). Here, tubulation and vesiculation requires extreme deformations to the limiting phagosomal membrane and is opposed by any tension on the vacuole (133). Tension comes in the form of osmotic pressure, generated because the solute concentrations of the phagosome can be higher than those of the cytosol. As solutes flux out of the phagosome, such pressure is relieved and the membrane becomes more pliable to these deformations by clathrin for example (150).

The exit pathways for solutes come in the form of solute carrier proteins (SLCs), and enormous family of transporters. While SLCs that flux amino acids and dipeptides out of the phagolysosome are the best appreciate in this regard (151–154), others that flux monosaccharides, metals, Na^+^ (155), Ca^2+^ (156), etc. will all be critical to the resolution process. The control of the efflux pathways is only recently becoming appreciated. The mammalian target of rapamycin (mTOR) and its associated complexes form major regulatory hubs that can both inhibit and stimulate amino acid efflux by SLCs (151, 152, 157) and even inhibit Na^+^ channels (155). We anticipate that the “phagosome-lysosome reformation” (PLR) will require exquisite control over its timing. For example, the organic solutes that contribute less osmotically to the compartment overall may be effluxed before more prevalent monovalent ions to ensure that the complete digestion and efflux of proteins and amino acids precedes the reformation of lysosomes. Of course, these ideas remain to be tested.

### Recycling Between the RPE and Photoreceptors

The photoreceptor outer segment (POS) grows as it is consumed by the RPE, marking a coupling between a highly anabolic cell type and a catabolic one (**Figure 4**). Throughout this review, we have emphasized the astonishing amount of material that the RPE must turnover per day. As 90% of the membrane shed in the POS derives from the photosensitive membranes of the internal discs, the total surface membrane of a phagosome is ~30 µm^2^ and the RPE consumes ~900 µm^2^ of surface membrane on average daily. It is not surprising that membrane-associated elements can be recycled by the RPE back to the photoreceptor (18). In some cases, the recycling pathways between the RPE and photoreceptors are well-established, especially for the retinoid cycle (158). As described previously, the phototransduction by opsins occur when the bound 11-*cis*-retinal undergoes isomerization to all-*trans*-retinal which in turns alters the conformation of the opsin. The return to the photosensitive receptor conformation requires the rejuvenation of the 11-*cis*-retinal from all-*trans*-retinal. Aside from the reduction of the all-*trans*-retinal to retinol, all reactions that support the retinoid cycle in fact take place in the RPE (159). The all-*trans*-retinol enters the RPE from circulation but a significant portion seems to be shuttled through the IPM (159) or by phagocytosis of the POS. The return of the 11-*cis*-retinal to the photoreceptor is in turn mediated by binding proteins, passive diffusion through the IPM, or direct transport.

The retinoid cycle represents just one of the recycling pathways between the cells. For example, the phospholipids of the photosensitive membrane discs of the POS are enriched in docosahexanoic acid (DHA), an omega-3 fatty acid. Remarkably, the retina can retain DHA even during long periods of dietary deprivation of omega-3 fatty acids (160). Careful experiments using radiolabeled DHA tracked the fatty acid through the POS and into the RPE and found that phagosomes are indeed packed with DHA to the same extent as the distal POS. Moreover, these types of experiments found that labelling in the RPE cytosol remains low and diffuse throughout extended (days long) periods of tracing, demonstrating that the DHA is quickly recycled back to the photoreceptors (22, 161).

While beyond the scope of this review, it should be mentioned that normal functions of RPE include maintaining an anti-inflammatory microenvironment and this is owed to the ongoing lipid metabolism of the phagosome. In particular, not all of the DHA is necessarily recycled back to the POS as is. The DHA supplied to the RPE can also be enzymatically converted to derive resolvins including neuroprotection D1 (NPD1) (162). NPD1 is a potent anti-inflammatory mediator that inhibits pro-apoptotic proteins and induces anti-apoptotic proteins, yielding cell-protective effects (162). On the other hand, the RPE can also contribute to pro-inflammatory pathways in certain contexts. Under sustained oxidative stress, for example, the RPE makes inflammatory cytokines including IL-6 and IL-1β and this can lead to AMD (163).

In addition to supplying the photoreceptor with retinal and DHA, the RPE sources the photoreceptors with glucose. To do so, the RPE expresses remarkably high levels of GLUT1 at their apical and basolateral surface (164). Glucose in the cytosol would normally be quickly converted for its use in glycolysis, but this is effectively suppressed in the RPE by the lactate that is produced and exported by the photoreceptor and because kinases that act on sugars are inhibited or expressed at sufficient low levels (164, 165). Conversely, such kinases (e.g. pyruvate kinase) are highly enriched in the photoreceptor making the cell highly glycolytic (164). When the RPE is made to consume more glucose, the neighboring photoreceptors become starved and ultimately degenerate. Using the same Glut1-based transport and others, we anticipate monosaccharides could be recycled from the RPE phagosome back to the IPM as well.

Finally, like other phagocytes, the RPE specialize in lipid/cholesterol efflux as emphasized by their robust expression and use of the cholesterol efflux regulatory protein ATP-binding cassette transporter A1 (ABCA1) (166). Here, ABCA1 shuttles cholesterol from the cytoplasm onto HDL. In the RPE, ABCA1 is targeted to both basolateral and apical membranes and can mediate efflux towards the subretinal space (i.e. the IPM) or, to a lesser extent, the choroidal space. Such efflux is more evident when the cells are given liver X receptor agonists or POS targets. Notably, the RPE is well-endowed with other cholesterol efflux proteins like SRB1 and ABCG1 (166). The RPE also metabolizes fatty acids found in the POS like palmitate to produce beta-hydroxybutyrate (167). Interestingly, photoreceptors were also recently found to use palmitate as an energy source through oxidative phosphorylation (168). The shuttling of palmitate or beta-hydroxybutyrate from the RPE to the photoreceptor as potential substates could therefore generally serve to maintain photoreceptor function (169).

It remains to be tested if other building blocks like sugars and amino acids are indeed recycled back to the photoreceptor once fluxed out of the RPE phagosome. Forms of direct transport, where photoreceptors and the RPE could be coupled by GAP junction proteins that support direct connections of the cytosol, are conceivable.

## Conclusion

The routine phagocytosis performed by the RPE of their photoreceptor neighbors serves as a reasonable guide for future investigations of heterocellular metabolic circuits as formed by tissue-resident phagocytes and the cells they prune and turnover. Unlike in the retina, other tissues can quite readily expand and contract with abrupt dietary changes or with developmental programs like involution. In these cases, the growth of resident populations of phagocytes are kept in check by the stroma: Well-defined paracrine growth factor circuits between stromal cells and macrophages can stabilize the ratios of these populations and this requires intimate contacts between the cells (170). Supporting mitogenesis in such circuits with nutrient supplies may be an overlooked feature of the coupling. A recent emphasis on cell-cell competition in tissues (171) may benefit from understanding the circuits, cell-cell sharing and recycling, and the metabolic ecosystems that ensure tissue homeostasis. Such studies would immediately lend themselves to an appreciation for how tumor-associated macrophages support the growth of lesions. Additionally, more emphasis on phagosome resolution and the handling of solutes in the endocytic pathway of phagocytes is will certainly lead to strategies that break or support such processes and cycles. The RPE-photoreceptor coupling is a well-defined Yin and Yang relationship but represents one of many more circuits to be uncovered.

## Author Contributions

WK and SF constructed figures and wrote/reviewed the manuscript. All authors contributed to the article and approved the submitted version.

## Funding

This work was supported by a CIHR Project Grant PJT #169180 (SAF). 

## Conflict of Interest

The authors declare that the research was conducted in the absence of any commercial or financial relationships that could be construed as a potential conflict of interest.
